# The Relationship between Depression Symptoms and Physical Activity in Children with Idiopathic Ventricular Extrasystoles

**DOI:** 10.3390/medicina60020213

**Published:** 2024-01-26

**Authors:** Rita Kunigeliene, Odeta Kinciniene, Vytautas Usonis, Sigita Lesinskiene

**Affiliations:** 1 Clinic of Children’s Diseases, Institute of Clinical Medicine, Faculty of Medicine, Vilnius University, 01513 Vilnius, Lithuania; 2 Clinic of Psychiatry, Institute of Clinical Medicine, Faculty of Medicine, Vilnius University, 01513 Vilnius, Lithuania

**Keywords:** depression, ventricular premature complexes, children

## Abstract

*Background and Objectives:* Depression in childhood often co-occurs with anxiety disorders and a range of somatic symptoms. Recent studies have identified physical activity as a target for preventing the onset of depression. However, idiopathic ventricular extrasystoles (VEs) in children are sometimes associated with somatic symptoms and limitations in physical activity. The occurrence of arrhythmia can also be distressing for children and their parents. This study was conducted to determine the relationship between symptoms of depression, physical activity, and somatic symptoms in children with idiopathic VE. *Materials and Methods:* This study of children with structurally normal hearts and VE was approved by the local ethics committee (no. 2021/10-1383˗859(1). The authors designed a questionnaire to assess symptoms, physical activity, and general well-being. As part of that, symptoms of depression were evaluated with a modified pediatric PHQ-9 (MP-PHQ-9) questionnaire, with scores ≤4 for no, 5–9 for mild, 10–14 for moderate, and ≥15 for severe depression. Children aged ≥12 years and parents who assessed their children’s condition completed the questionnaires. All children also underwent 24-h electrocardiography and echocardiography to evaluate arrhythmia frequency and cardiac condition. *Results:* Questionnaires were completed by 60 children’s parents and 39 children (≥12 years old). The median children’s age was 13 years. Palpitations were experienced by 26 (43.3%), chest pain by 13 (21.7%), and exercise intolerance by 15 (25%) children. All patients had normal ventricular function and hemodynamically normal hearts. The median score of the MP-PHQ-9 completed by parents was 2, and by children was 4. The median VE frequency was 4.77 (0.1–32.77) % per 24 h. We found that 31 (51.7%) children engaged in extra-sports participation with a median time of 3.75 h per week. Eleven of the children were suspended from sports. There was no significant difference between VE frequency and MP-PHQ-9 scores. Higher MP-PHQ-9 scores were noted for symptomatic children who engaged in <5 h per week of physical activity. *Conclusions:* Higher depression scores were found for children with somatic symptoms than those without symptoms. Children who were physically active for less than 5 h per week also had higher depression risk scores than those who were more active. Our research has shown that parents underestimate the signs of depression in their children.

## 1. Introduction

Ventricular extrasystoles (VEs) are one of the most common arrhythmias occurring in patients with heart abnormalities as well as in healthy children [1]. Ventricular extrasystoles in patients with structural heart disease are often associated with a risk of ventricular tachycardia [2]. However, when VEs occur in patients with structurally normal hearts, these so-called idiopathic VEs are mostly considered benign [2]. It is estimated that 18–50% of healthy children have VE detected on ambulatory monitors [3]. Although idiopathic ventricular extrasystoles in children are considered benign, about one-third of patients experience somatic symptoms [4] and face limitations in physical activity. The identification of VE and its potential impact on fitness for sport is a frequent topic of debate involving physicians from different specialties [5]. For precautionary reasons, the affected children are often unjustifiably disqualified from sport participation, even at a non-competitive level [5]. In support of this, recent studies have shown that patients with VEs have lower exercise capacity than healthy children [6]. However, it is not clear whether the reduction in exercise capacity is due to exercise restriction or due to the symptoms or arrhythmia itself.

Physical activity is likely to have positive psychosocial outcomes [7]. A systematic review by Rodriguez-Ayllon et al. showed a statistically significant negative association between physical activity and depression (28.5 of 43 studies, 66.3%), stress (6 of 6 studies, 100%), negative affect (3 of 4 studies, 75%) and total psychological distress (5 of 8 studies, 62.5%) [8]. Increasing physical activity is recognized as a potential target for preventing the onset of depression [9] and for reducing the symptoms of depressive and anxiety disorders, especially in those affected by mild to moderate depression [10]. According to the review by Biddle et al., higher levels of exercise were significantly associated with lower depression rates among young people [7]. Moreover, exercise significantly reduced depressiveness among participants with clinical depression and depression resulting from mental illness [7]. Depression is a major global mental health problem worldwide [11]. Depression is a mood disorder that affects the emotional, cognitive, and behavioral dimensions and negatively affects the whole functioning of the person [12]. It is common and often recurrent, with a global prevalence of 4% in school-aged children, 7.5% in adolescents, and 4.4% in the general population [11,13,14]. Depressive symptoms often co-occur with anxiety disorders [15] and a range of somatic symptoms and signs, particularly early in life. This reflects that there is an association between somatic complaints and mental disorders. As depression is very often associated with other disorders, it is no longer clear what is the cause and what is the effect.

Besides this, the problem of having an arrhythmia can be distressing for children and their parents. According to Porcedda et al., the follow-ups for these children can last for a decade [5]. During the process of arrhythmia evaluation and monitoring, these patients must attend many outpatient and/or inpatient care visits, which is stressful for patients and their parents. Heart rhythm disturbances must be sensitively handled because most parents and patients associate arrhythmias with a risk of sudden cardiac death. Accordingly, children with heart rhythm problems are at risk of parental overprotection. Overprotective parents tend to restrict physical activity, and previous work has found an association between overprotective parenting styles and anxiety and depression in children [15].

Our study was conducted to determine the relationship between physical symptoms, signs of depression, and physical activity in children with idiopathic VE and to compare patients’ and their parents’ ratings of children’s somatic and depressive symptoms.

## 2. Materials and Methods

### 2.1. Participants and Procedure

This prospective study of children with structurally normal hearts and VE was approved by the Regional Ethics Committee No. 2021/10-1383˗859(1). The inclusion criteria were 3–17 years old children with and/or more than 5% VE per 24 h and/or paired and/or multiform VE, for whom we had signed informed consent (by parents or guardians of each participant, or/and by children if aged ≥12 years). The exclusion criteria were diagnoses of hemodynamically significant heart diseases, channelopathies, or refusal to participate in the study. This cross-sectional study was performed in the Vilnius University Hospital Santaros Clinics from 1 January 2022 to 30 August 2023. The authors designed questionnaires to assess a patient’s age, biological gender, symptoms, physical activity, and depressive symptoms. The relevance and validity of the questionnaire were tested in a local pilot study. Children aged ≥12 years old and parents who assessed their children’s condition completed the questionnaires. All children also underwent a 24-h electrocardiogram and echocardiography to evaluate arrhythmia frequency and their cardiac condition. During the echocardiography studies, we evaluated patients’ hearts for possible structural, septal, valvular, or myocardial abnormalities and assessed the left ventricular function using the Simpson biplane method. Normal left ventricular function was considered to be more than 55%. Minor valvular leaks and small shunts at the *foramen ovale* site were considered hemodynamically insignificant. In the 24-h electrocardiogram, we assessed the total number of VEs, the number of paired, bigeminy, and trigeminy VEs, and the different morphologies of the VEs over 24 h. We evaluated the results of all 24-h ECGs performed in all subjects. We included the maximum number of extrasystoles reached and the number of extrasystoles in the latest 24-h ECG with the latter recorded ≤1 month before the completion of the questionnaires and the echocardiographic examinations.

### 2.2. Measurements

#### 2.2.1. Assessment of Symptoms

The questionnaires included questions on symptoms such as chest pain, palpitations, syncope/fainting, weakness, fatigue, dyspnea, and exercise intolerance. Respondents rated the frequency of the symptoms and the relationship between the symptoms and physical activity as never/rarely/sometimes/often/very often. The answers sometimes/often/very often were scored as having symptoms or relationships, respectively.

#### 2.2.2. Assessment of Physical Activity

We assessed the amount of time per week spent on physical activity. The questionnaires included questions about the amount of time per week spent in physical education at school and extra time spent on sport activities. We divided the children into groups according to the amount of time spent engaging in physical activity: up to 6 h per week—leisure activity, 6–10 h per week—competitive sports, and more than 10 h per week—elite sports.

#### 2.2.3. Assessment of Depressive Symptoms

Symptoms and depression were evaluated using a modified pediatric version of the Patient Health Questionnaire (PHQ-9). The PHQ-9 is a well-validated, criterion-based measure for diagnosing depression, assessing severity, and monitoring treatment response [9]. The PHQ-9 scale evaluates these items: (1) Thoughts that you would be better off dead/hurting yourself; (2) Moving or speaking slowly/being fidgety or restless; (3) Trouble concentrating; (4) Feeling bad about yourself/that you are a failure/letting people down; (5) Poor appetite or overeating; (6) Feeling tired/having little energy; (7) Trouble falling asleep/staying asleep/sleeping too much; (8) Feeling down/depressed or hopeless; (9) Little interest or pleasure in doing things [16]. Each of these nine items scored on a scale of 0 (not at all) to 3 (almost every day). The PHQ-9 score can range from 0 to 27 [17]. The overall score is interpreted as follows: ≤4—no signs of depression; 5–9—mild depression; 10–14—moderate depression; ≥15—severe depression. A PHQ score of ≥10 has been reported to have both a sensitivity and specificity of 88% for major depression [17]. A recent study suggested that the PHQ-9 with a critical score of 10 had the best sensitivity (Se = 0.91) and specificity (Sp = 0.76) among adolescents aged 10–19 years [16].

### 2.3. Statistical Analysis

Statistical analysis was performed using R software (version 4.2.2). Nominal variables were tested for a normal distribution with the Shapiro-Francia test. Normally and non-normally distributed variables were presented as the mean and standard deviation or median, minimum, and maximum values, respectively. Categorical variables were presented as numbers (percentages). Comparisons of continuous variables between two groups were carried out using the Student’s *t*-test or chi-square test according to the distribution. Comparisons of continuous variables between different groups were performed using one-way analysis of variance (ANOVA) or the Kruskal-Wallis test according to the distribution. Comparisons of categorical variables were conducted using the chi-square or Fisher’s exact test. The correlation between variables was evaluated with Pearson’s correlation coefficient (r) or Spearman’s rank correlation coefficient (r_s_) according to the distribution. A *p*-value ≤0.05 was considered statistically significant.

## 3. Results

### 3.1. Characteristics of the Patients

**Scheme 1:**
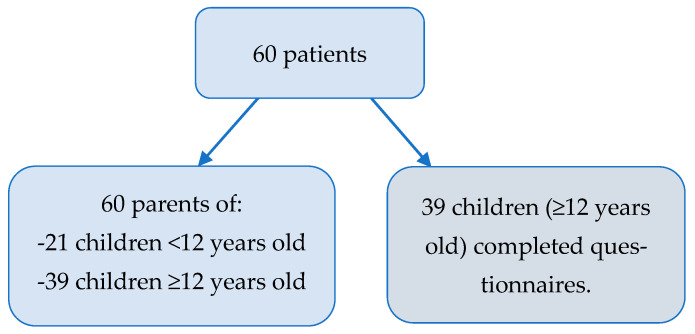
Questionnaires completion scheme.

Questionnaires were completed by 60 parents of children and 39 children aged >12 years (Scheme 1).

The median age of the patients was 13 (5–17) years.

The latest tested VE count median was 4.77% (0.1–32.77%) per 24 h, while the maximal achieved VE count median was 6.73% (0.34–34.47%) per 24 h. All children had hemodynamically normal hearts. The median left ventricular function assessed using Simpson’s biplane method was in the normal range at 61.5% (55.4–70.7%).

The main characteristics of the patients are shown in Table 1.

### 3.2. Symptoms

Twenty-seven patients were asymptomatic. Parents rated the MP-PHQ-9 score of their asymptomatic children statistically significantly better than those with chest pain (2 vs. 5, χ^2^ = 5.72, df = 1, *p* = 0.02) and dyspnea (2 vs. 11, χ^2^ = 11.14, df = 1, *p* = 0.0008). There were no MP-PHQ-9 scores higher than 10 in asymptomatic children.

The most common symptom was palpitations. Chest pain was experienced more by older children (median age—16 years vs. 12 years old; χ^2^ = 6.38, df = 1, *p* = 0.01) with no difference in other symptoms based on age.

Patients with symptoms demonstrated significantly higher median children’s MP-PHQ-9 scores than those without symptoms: palpitations 8 vs. 3 (χ^2^ = 6.13, df = 1, *p* = 0.01); chest pain 10 vs. 3 (χ^2^= 6.6177, df = 1, *p* = 0.01); dyspnea 12 vs. 3 (χ^2^ = 8.88, df = 1, *p* = 0.003); exercise intolerance 10 vs. 3 (χ^2^= 4.69, df = 1, *p*-value = 0.03), fatigue 9.5 vs. 3 (χ^2^ = 10.712, df = 1, *p* = 0.001). There was no difference in children’s MP-PHQ-9 scores between children with syncope/fainting and other children (2 vs. 4, χ^2^ = 0.28, df = 1, *p* = 0.6).

Adolescents with more than two symptoms were more likely to have a higher MP-PHQ-9 score than their peers (9 vs. 3, χ^2^ = 8.32, df = 1, *p* = 0.004).

### 3.3. Physical Activity

The median total time of physical activity was 3.75 (1.5–28.75) h per week. The median time spent engaging in physical activity during school time was 2.25 (1.5–7.5) h per week. Thirty-one (51.7%) children participated in extra sports with a median extra sports activity time of 3.75 (0.75–25) h per week.

Patients without symptoms were more physically active than their symptomatic peers (median activity time 3.75 vs. 1.5 h/week; χ^2^ = 5.37, df = 1, *p* = 0.02). In addition, patients with more than two symptoms were less physically active than those with fewer symptoms (1.5 vs. 3.75 h/week; χ^2^ = 6.86, df = 1, *p* = 0.009).

**Figure 1:**
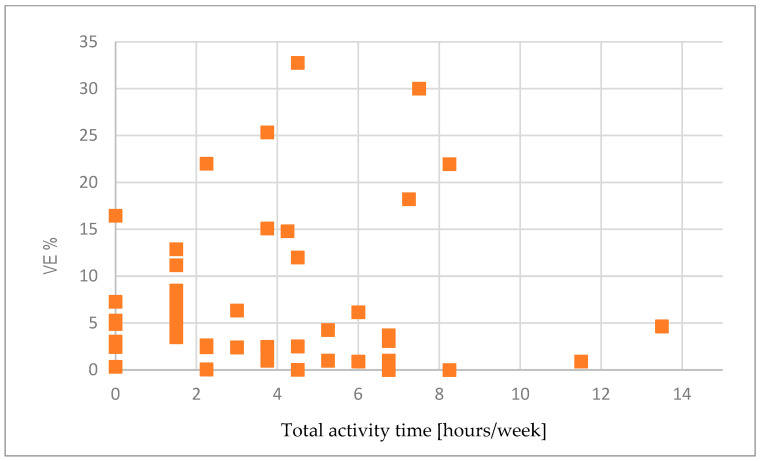
Comparison between activity time and latest VE frequency % per 24 h (r_s_ = −0.2; *p* > 0.05).

The physical activity time was not correlated with the latest frequency of VE (r_s_ = −0.2; *p* > 0.05) (Figure 1).

The physical activity of 11 (18.3%) patients was suspended. However, one of those patients continued to participate in competitive sports.

### 3.4. Depressive Symptoms

The median score of the MP-PHQ-9 completed by parents was 2 (0–16), and by children, it was 4 (0–18). Parents underestimated their children’s depressive symptoms (χ^2^= 7.1, df = 1, *p*-value = 0.008).

Three patients had MP-PHQ-9 scores greater than 15, and eight patients had MP-PHQ-9 scores higher than 10; two of them were suspended from physical activity, and two of them participated in extra leisure sports.

**Figure 2:**
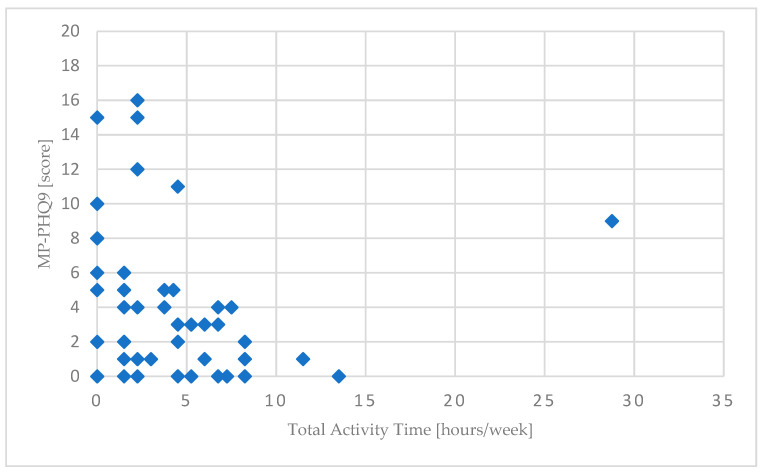
The relationship between parents’ MP-PHQ-9 scores and total physical activity time (r_s_ = −0.29; *p* > 0.05).

Patients with MP-PHQ-9 scores higher than 10 engaged in physical activity for a total of fewer than 5 h per week (Figure 2).

**Figure 3:**
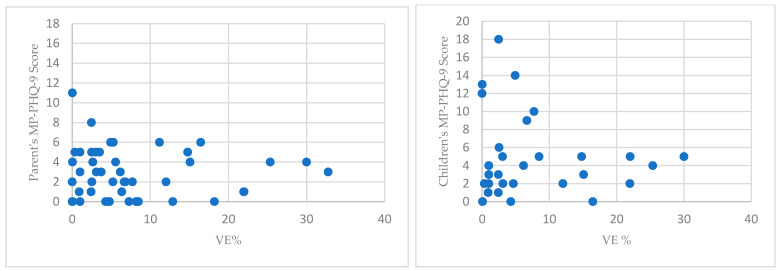
The relationship between parent’s and children’s MP-PHQ-9 scores and VE% (r_s_ = 0.02 and 0.06; *p* > 0.05).

There were no significant differences between the counts of VE, and the questionnaire results (*p* > 0.05) (Figure 3).

The comparison between parents’ and children’s median MP-PHQ-9 scores is shown in Table 2.

## 4. Discussion

Children with chronic health conditions often have emotional and mood disorders. Although pediatric idiopathic VE is considered benign, in our study, 8.3% of patients showed signs of moderate depression, and 5% showed signs of severe depression. According to Shorey et al.’s meta-analysis of the general population of adolescents, 8% had major depressive disorder, and 34% had depressive symptoms [18]. As such, the prevalence of depressive signs noted in our study is close to the prevalence of depression in the general population.

Early identification of depressive symptoms is associated with better recognition of the problem and more effective treatment. The role of the family in recognizing early signs of depression is unquestionable. Family members are very important as direct and indirect models of emotional understanding and communication [19]. However, our research has shown that parents underestimate the signs of depression in their children.

Many studies in children and adolescents have shown an association between depression and complaints of somatic symptoms [20]. For example, fatigue is strongly associated with depression [19]. On the other hand, other types of somatic symptoms seem to be more strongly associated with anxiety (e.g., chest pain) [20]. In our study, the children who experienced symptoms of palpitations, chest pain, dyspnea, and exercise intolerance demonstrated significantly higher depression scores. In addition, more somatic symptoms were associated with higher levels of depression in our study.

Data from different authors have shown that between 23% and 48.5% of children with VE experience somatic symptoms [4,21,22], such as palpitations, fainting, fatigue, chest pain, or syncope [22]. In our study, 45% of the children had symptoms. This can be explained by the assessment of somatic symptoms according to the frequency of their occurrence. In our study, palpitations were the most frequent symptoms.

Physical activity plays an important role in the prevention of mental health problems [19]. Meta-analyses demonstrate that participation in physical activity leads to a significant reduction in symptoms of depression in people with established depression, suggesting a role for physical activity as part of therapeutic interventions [20]. A recent population meta-analysis found that people with high levels of physical activity had 17% lower odds of depression than people with low levels of physical activity [23,24]. Low physical activity is also associated with a higher risk of depression [24]. In our study, adolescents who were active for fewer than 5 h per week were at risk of moderate depressive symptoms. Meanwhile, there was no evidence of moderate or severe depression in the competitive and elite sports groups. The World Health Organization recommends 60 min of moderate-to-vigorous physical activity per day for 5- to 17-year-old children [25]. This equates to 5–7 h per week [25]. Only 40% of the children in our study met the World Health Organization guidelines for physical activity.

A key strength of our study is its prospective nature. We assessed the association between somatic complaints, depression scores, and physical activity time in children with ventricular extrasystoles, which are usually considered benign. We scored somatic symptoms according to their frequency of occurrence. The amount of physical activity was assessed in hours per week, so we did not assess the exertion during physical activity using diaries, activity scales, or pedometers. Meanwhile, a key limitation of our study is that our research was observational, and the findings do not allow us to define the cause and effect between reduction in physical activity and somatic and psychological symptoms. Our study also has other limitations. For instance, the number of patients included is limited, and the observation period is short, thus leaving a possibility of error. Furthermore, we did not conduct a double-blind study, so the results may be susceptible to selection bias. Finally, due to the limited number of patients with depressive symptoms in our sample, the risk factors could not be properly assessed.

It is still uncertain how somatic symptoms and depression determine the intensity of physical activity in patients with idiopathic ventricular extrasystoles. It is not clear whether somatic symptoms and physical activity are a cause or a consequence of depression. However, it is important that an association was found between depression, somatic symptoms, and physical activity.

Further research should focus on improving our understanding of the relationships between physical activity and depressive signs in patients with idiopathic ventricular extrasystoles. Defining their linkages may support the development of suggestions for more effective approaches to how we regard these children’s capacity for physical activity, which will represent an important step forward, given the possibility that physical activity can prevent the onset of depression. The essential factors to consider are patients’ personal characteristics, their relationships with their parents, parental anxiety, and overprotection levels. Furthermore, physical activity should be assessed using wearable devices and detailed questionnaires.

## 5. Conclusions

In our study, children who were physically active for fewer than 5 h per week had higher depression risk scores than those who were more active, while patients with somatic symptoms had higher depression scores than those without such symptoms. Meanwhile, our research has shown that parents underestimate the signs of depression in their children. Therefore, idiopathic ventricular extrasystoles, limitations in physical activity, and depression may be connected in ways that would help clinicians to understand, which highlights the need for further research on their linkages.

## Figures and Tables

**Table 1:** Characteristics of the patients.

Variable	N (%)
**Gender**	
Female	33 (55%)
**VE characteristics**	
Monomorphic	50 (83.3%)
Multiform	10 (16.7%)
Bigeminy	22 (36.7%)
Trigeminy	3 (13.3%)
Paired VE	21 (35%)
**Symptoms**	
Chest pain	13 (21.7%)
Palpitations	26 (43.3%)
Syncope/fainting	5 (8.3%)
Weakness	13 (21.7%)
Fatigue	18 (30%)
Dyspnea	8 (13.3%)
Exercise intolerance	15 (25%)
**No symptoms**	27 (45%)
1 symptom	12 (20%)
≥2 symptoms	21 (35%)
**Physical activity**	
Suspended	11 (18.3%)
Leisure (<6 h/week):	34 (68%)
<4 h/week	26 (52%)
4–6 h/week	8 (16%)
Competitive (6–10 h/week)	13 (26%)
Elite (≥10 h/week)	3 (6%)
**Extra sports**	31 (51.7%)
**MP-PHQ-9**	
Parents’ answers	60
Score < 10	54 (90%)
Score ≥ 10	6 (10%)
Children’s answers	39
Score < 10	31 (79.5%)
Score ≥ 10	8 (20.5%)

**Table 2:** Comparison between parents’ and children’s median MP-PHQ-9 scores.

	Median MP-PHQ-9 Scores—Parents (N-60)	Median MP-PHQ-9 Scores—Children (N-39)
**Symptoms**		
Chest pain (N-13)	5	10
Palpitations (N-26)	3	8
Syncope/fainting (N-5)	0	2
Weakness (N-13)	6	10
Fatigue (N-18)	3	9.5
Dyspnea (N-8)	11	12
Exercise intolerance (N-15)	5	10
No symptoms (N-27)	3	4
≥2 symptoms (N-21)	5	9
**VE characteristics**		
Multiform (N-10)	2	2.5
Bigeminy (N-22)	2.5	3
Trigeminy (N-3)	3.5	4.5
Paired VE (N-21)	2	3
**Physical activity**		
Suspended (N-11)	6	5
Leisure (<6 h/week) (N-34)	2	4
Competitive (6–10 h/week) (N-13)	1	4
Elite (≥10 h/week) (N-3)	1	2

## Data Availability

On request, the corresponding author will provide the datasets produced and/or analyzed during the current study.
